# Beyond the hype: a call for comprehensive evidence on the economic and health outcomes of digital and data technologies

**DOI:** 10.1093/oodh/oqae041

**Published:** 2024-12-02

**Authors:** Laura Edison Kallen, Sarah Skye Yoden, Tara Herrick, Wuleta Lemma, Marelize Gorgens

**Affiliations:** Center for Digital and Data Excellence, PATH, 455 Massachusetts Avenue NW, Suite 1000, Washington, DC 20001, USA; Department of Health Policy and Management, Harvard University, 677 Huntington Avenue, Boston, MA 02115, USA; Market Dynamics, PATH, 2201 Westlake Avenue, Suite 200, Seattle, WA 98121, USA; Wollo University, 1145 Dessie, Ethiopia; Human Development Practice Group, The World Bank, 1818 H Street, Washington, DC 20433, USA

**Keywords:** digital health interventions, impact, cost-effectiveness, evidence, health outcomes, financing

## Abstract

Digital and data technologies are increasingly being implemented to meet a range of global health needs, including strengthening health systems, improving efficiency and advancing universal health coverage. However, there is limited quantitative evidence and few standardized methodologies for analyzing the health impact and cost-effectiveness of digital and data technologies in health systems in low- and middle-income countries. Without such evidence, development partners, patients, civil society and government leaders have a limited basis from which to incentivize, justify and allocate funding for sustained investments in digital and data technologies, which in turn impacts their sustainability. Dedicated funding and planning for such efforts can help decision-makers hold digital health providers accountable and improve the allocation of limited resources.

**RESUMEN:**

Las tecnologías digitales y de datos se están implementando cada vez más para satisfacer distintas necesidades de salud a nivel global, incluyendo fortalecer los sistemas de salud, mejorar la eficiencia y propiciar la cobertura universal de salud. Sin embargo, hay evidencia cuantitativa limitada y pocas metodologías estandarizadas para analizar el efecto en la salud y la efectividad de costos de las tecnologías digitales y de datos en los sistemas de salud en países de ingresos bajos y medios. Sin tal evidencia, los socios de desarrollo, los pacientes, la sociedad civil y los líderes gubernamentales tienen una base limitada para crear incentivos, justificar y asignar fondos para inversiones continuas en tecnologías digitales y de datos, lo cual a su vez afecta su sustentabilidad. La asignación de fondos y la planificación dirigidas para tales esfuerzos pueden ayudar a las personas encargadas de tomar decisiones a confiar la responsabilidad a los proveedores de salud digital y a mejorar la asignación de recursos limitados.

**RESUMO:**

As tecnologias digitais e de dados estão a ser cada vez mais implementadas para responder a uma série de necessidades globais de saúde, incluindo o reforço dos sistemas de saúde, a melhoria da eficiência e o avanço da cobertura universal de saúde. No entanto, existem poucos dados quantitativos e poucas metodologias normalizadas para analisar o impacto na saúde e a relação custo-eficácia das tecnologias digitais e de dados nos sistemas de saúde dos países de baixo e médio rendimento. Sem essas provas, os parceiros de desenvolvimento, os doentes, a sociedade civil e os líderes governamentais têm uma base limitada para incentivar, justificar e atribuir financiamento para investimentos sustentados em tecnologias digitais e de dados, o que, por sua vez, afeta a sua sustentabilidade. O financiamento e o planeamento dedicados a esses esforços podem ajudar os decisores a responsabilizar os fornecedores de saúde digital e a melhorar a afetação de recursos limitados.

**RÉSUMÉ:**

Les technologies numériques et de données sont de plus en plus utilisées pour répondre à une série de besoins de santé mondiaux, notamment le renforcement des systèmes de santé, l’amélioration de l’efficacité, et la promotion de la couverture sanitaire universelle. Cependant, il existe peu de données quantitatives et peu de méthodologies standardisées pour analyser l’impact sur la santé et la rentabilité des technologies numériques et de données dans les systèmes de santé des pays à revenu faible et intermédiaire. Sans ces preuves, les partenaires de développement, les patients, la société civile et les dirigeants gouvernementaux disposent d’une base limitée pour encourager, justifier et allouer des fonds aux investissements durables dans les technologies numériques et de données, ce qui a à son tour un impact sur leur durabilité. Un financement et une planification dédiés à ces efforts peuvent aider les décideurs à demander des comptes aux prestataires de santé numérique et à améliorer l’allocation de ressources limitées.

## INTRODUCTION

The transformation of health systems in low- and middle-income countries (LMICs) using digital and data technologies is widely recognized as having tremendous potential to accelerate progress toward universal health coverage. The World Health Organization Global Strategy on Digital Health 2020–2025 [1] states:

[…] there is a growing consensus in the global health community that the strategic and innovative use of digital and cutting-edge information and communications technologies will be an essential enabling factor toward ensuring that 1 billion more people benefit from universal health coverage, that 1 billion more people are better protected from health emergencies, and that 1 billion more people enjoy better health and well-being.

Based on this promise, donors and country governments have invested heavily in digital health interventions (DHIs) to meet a wide range of global health needs. DHIs are being implemented to improve the quality and coverage of patient care, decrease the workload of health care workers, strengthen health systems to increase resiliency against pandemics and climate change disruptions, improve health system efficiency and advance universal health coverage.

While DHIs are universally recognized as an increasingly necessary part of efforts to modernize health systems, there is a significant lack of quantitative evidence on the impact and cost-effectiveness of DHIs. Much of the existing evidence on the health, human and financial impact of DHIs is qualitative and informal, uses a non-standardized methodology and focuses on intermediate outputs rather than health outcomes. While important, without complementary quantitative cost-effectiveness and impact data, the existing evidence has limited value for countries seeking to inform national health policies and figure out how to best implement programs [2].

We believe this evidence gap exists for three primary reasons. First, digital tools are rarely the only intervention being implemented. They are more often part of a broader intervention package. It can therefore be difficult to isolate a digital tool’s impact and cost-effectiveness. Relatedly, digital solutions have often been piloted or implemented in silos or in single-use, vertical applications, which can limit the true understanding of costs and benefits at scale. Even digital tools built for just one use can have cross-cutting benefits across other parts of the health system.

Second, measuring the value of DHIs can be technically challenging due to a lack of clarity around best-in-class digital tool costing methods, the many different methods for evaluating impact, and a lack of best practices in defining counterfactuals for digital deployments [3]. However, a growing body of evidence and methodologies for analyzing the cost, impact and cost-effectiveness of DHIs—many of which are outlined in this supplement —shows that conducting these analyses is feasible and affordable despite the technical challenges.

Third, there is a lack of dedicated funding for determining DHI costs and designing and running impact evaluations and other types of evaluations and operations research. The lack of funding may be attributable to a lack of awareness of the need for such evaluations or research among the digital health community—including funders, implementers and developers. They may be unaware or skeptical of the value that this research would add to decisions about funding allocations to digital health in resource-constrained environments where governments sometimes must make difficult decisions between funding immediate service delivery and funding longer-term foundational digital investments.

As this commentary will argue, DHI impact and implementation evidence is in high demand from—and needed by—both country decision-makers and donors to prioritize future allocations around digital health. To meet this demand, analyses assessing the cost and value of DHIs should become a standard in the field.

## COUNTRIES NEED EVIDENCE FOR INFORMED DECISION-MAKING ON DHIs

When a government stakeholder has an opportunity to introduce any new intervention as part of a country’s national health system—whether a new vaccine or a new electronic immunization registry technology—they must weigh many considerations. What problem does it solve? What value would the intervention have to the health system and to health outcomes? Is there demand, and would health workers or the public use it? Will it save money? How will it disrupt or improve existing ways of doing it? And—often a key consideration—what will it cost, and can the country’s budget support its use year after year?

In order to introduce and sustain investments in digital health, the intervention needs to be costed, justified and budgeted. Decision-makers need evidence to be able to make informed decisions on how to use limited resources to achieve the most health impact.

Many health intervention impact and cost assessments have standard approaches and comparative tools, such as the Tufts Cost-Effectiveness Registry [4], the Disease Control Priorities program [5], or efforts of health technology assessment agencies like the Health Intervention and Technology Assessment Program (in Thailand) [6] or National Institute for Health and Care Excellence (in the United Kingdom) [7]. If the intervention in question is, for example, a vaccine, decision-makers usually use a wide range of evidence and tools to analyze the cost, cost-effectiveness and health impact to inform their decision-making. The vaccine will cost a certain amount to add to the country’s vaccination schedule and will presumably avert a certain amount of disease and/or death. These figures allow for standardized estimates of impact and cost-effectiveness (e.g. cost per disability-adjusted life-year [DALY] or quality-adjusted life-year [QALY]), and in turn, these estimates allow for straightforward comparisons of one vaccine or intervention with another.

If the intervention is a DHI, the question of potential impact and cost-effectiveness is more complicated. Implementing a DHI—and/or building the digital infrastructure necessary for countries to support and maintain the technology—will cost a certain amount of money, but the costs may be shared by multiple stakeholders and may include hidden or unexpected costs, leading to a lack of clarity around budget allocations. At a presentation at the Asia e-Health Informatics Network 2022 Annual Meeting, an Asia-based country government stakeholder shared this reflection on the lack of clarity around digital health costs:

The question is always when we request budget for new tools, the Ministry of Finance will ask, what is the return on investment? What is the total cost of ownership? As people from the medical background, we do not know how to answer that.

To gain more insights from decision-makers, the authors of this commentary sent a survey to a sampling of country and regional digital health decision-makers. One respondent, Dr Fazilah Shaik Allaudin, State Health Director, Penang State Health Department of the Ministry of Health, Malaysia, shared this reflection about DHIs:

The biggest challenge is to show quantitative benefits—cost savings, cost-effectiveness, return on investment, etc. I would think this is one of the issues that makes funding for digital interventions difficult or not given higher priority compared to drugs, medical equipment, and facilities.

Given the limited budgets of LMIC governments, questions of cost are often a key consideration for country decision-makers. But traditional measures of cost-effectiveness—such as DALYs and QALYs—may not be suitable for quantifying and comparing the value of DHIs, as Kolasa and Kozinski [3] have argued. Because many DHIs improve health ‘system’ outcomes and not health outcomes directly, alternative metrics to DALYs or QALYs may be needed. Another factor, however, is the value of information—i.e. there is a maximum amount that a decision-maker may be willing to pay for information depending on the potential impact of the decision. If, for example, a country government is already very confident about a DHI investment decision, the willingness to pay for analyses will be relatively low. In these circumstances, this supplement provides clues on how to perform less costly analyses, such as modeling potential health impact rather than measuring the impact in a clinical study.

When asked what types of results would be most helpful for analyzing programs, Jean-Philbert Nsengimana, Chief Digital Advisor for the Africa Centres for Disease Control and Prevention, responded:

In practice, we use all types of impact measures, depending on context. However, if we had to choose one, we would go for DALYs averted to quantify the overall disease burden reduction and align with core public health goals and equity principles. DALYs have the added advantage of the availability of global benchmarks and cost-effectiveness thresholds. Measuring the cost per DALY averted allows us to understand and prioritize high-impact, cost-effective interventions across diverse health areas.

Evidence on health impact is also important for countries. The DHI will, presumably, improve health and save lives, but often in a more indirect way compared to something like a vaccine. For example, digital decision-support tools may improve care outcomes by increasing adherence to current clinical guidelines and improve coverage of effective medical interventions. DHIs may also improve the efficiency and effectiveness of the health system by freeing up health workers’ time and potentially reducing costs. These indirect benefits can be more difficult to measure, but the evidence is necessary for countries to justify a budget decision and to build support for the investment. In response to this topic, Ada Mohammed Al-Qunaibet, Chief Officer of Public Health Intelligence at the Public Health Authority of Saudi Arabia, reflected:

It's not just about how you are saving money at the hospital level. It is about the whole ecosystem of human beings. How does having better data using digital health help you decrease individuals’ costs and the country’s overall spending?

The need for evidence-based decision-making does not end with a country’s initial decision to implement. Data gathered through continued monitoring and evaluation throughout the lifecycle of a DHI can help build demand and support for an intervention among users, identify any issues, make adjustments as needed and generate sustained funding for adaptation, expansion, improvements and ongoing operating expenses. One story from Nsengimana reflects the difference that having such evidence could have made for a country’s digital health program:

In one of our Member States, the government partnered with an international digital health service provider to offer virtual consultations and health services to underserved communities. Initially, the program showed promising results in pilot areas—increasing access to care and reducing travel times and associated costs for patients. However, as the scaling of this intervention nationwide was evaluated, robust impact evidence became crucial for informed decision-making. Rigorous studies assessing health outcomes, cost-effectiveness, and user satisfaction across different regions would have been extremely valuable. For instance, evidence indicating the solution led to early diagnoses and improved management of chronic conditions like diabetes or hypertension could have justified expanding it as a priority intervention. Cost-effectiveness data comparing the platform’s costs to DALYs averted may have allowed us to rank it favorably against other health investments. Additionally, systematic monitoring of service usage, dropout rates, and feedback from diverse user groups could have identified barriers to adoption or population segments being underserved. This evidence could have then guided modifications to the technology, deployment strategies, or accompanying services to enhance accessibility and sustainability before nationwide scale-up. Partly as a result of this lack of evidence, the initiative collapsed.

More high-quality evidence of the cost of DHIs also helps with the creation of costed national digital health strategies, which in turn help ensure money for monitoring and evaluation, creating a positive feedback loop. All decision-makers interviewed agreed that a costed national digital health strategy and implementation plan would help their country allocate funding for monitoring and evaluation of interventions.

## FUNDERS NEED DHI IMPACT EVIDENCE TO SUSTAIN INVESTMENTS

From a funder or donor perspective, impact evidence is currency. They owe it to their governing bodies, individual donors, and/or taxpayers to show that their investments are achieving their intended results—improving health, saving lives and increasing health system efficiencies in costs, time and human resources. While most funders have some requirements for monitoring and evaluation, the type of evaluation is often left up to the funded organization.

The authors of this commentary also interviewed and sent a survey to a variety of representatives from different digital health funding organizations. Funders noted several challenges to conducting impact and cost assessments, including quality of evidence, a lack of time and limited funding. In response to an inquiry on challenges, Samuel Battistoli of the Dovetail Impact Foundation noted the variety in quality of evidence as well as limited time to conduct assessments:

We provide unrestricted core funding to our partners. If they elect to use this funding for impact evaluations, we would be quite excited. [However,] the quality of organizations’ evidence bases may vary widely. Do they exist at all? Do they include studies with confounding results? Do they include studies of related but not identical interventions or technology? Dollar-denominated studies of the contribution of digital health to health sector efficiency are useful to us but seem to be relatively rare […] though we recognize that there are many demands on stakeholders’ time.

Despite these challenges, there is a huge demand for more evidence on the impact and cost of DHIs among funders. The US Agency for International Development (USAID) Center for Innovation and Impact reflected that long-range impact evidence of large-scale digital programs remains an unmet need. Melissa Miles, a program officer at the Gates Foundation, noted:

We recognize the potential of digital in health and have been inspired by bright spots but recognize the current dearth of evidence. This is why we focus our support on evidence generation for promising country-led innovations that seek to improve access to or quality of primary healthcare for underserved populations. […] Ultimately, we are looking for innovations that can demonstrate a tangible contribution to health benefits or improvements in efficiency, whether cost savings, worker productivity, etc. Our rigorous focus on evidence is in acknowledgment of countries’ overwhelming number of priorities and reflects a desire to support decision-making about how best to allocate resources.

While all types of evidence can be useful to funders, they are more likely to prioritize health impact or health care access data. Like country decision-makers, however, funders can use different types of evidence at different stages of an intervention project. Battistoli of the Dovetail Impact Foundation commented on prioritizing health impact data but also valuing cost data:

Health impact data, when available, is of primary interest. Improved access to care of a defined, superior quality is easier to demonstrate and often sufficient to our needs. Either in the absence of or alongside of health impact or care access data, efficiency and cost savings data expressed in dollar terms are of major value. We’re excited about the dawning prospect of being able to see the social return on investment (SROI) of digital interventions as relates to a sustainability threshold, though we’ve not yet encountered this in practice. We also take note of cost per DALY or QALY averted.

High-quality evidence can have a large impact on sustaining financial support for a program. For example, Battistoli noted that, after receiving the results of a USAID-funded randomized clinical trial for a DHI, the Dovetail Impact Foundation provided a surge grant that increased their annual funding to a partner by 500%.

## CALL TO ACTION: INCREASING INVESTMENT IN DHI MONITORING AND EVALUATION

It is estimated that the global digital transformation of health systems will require a total of $12.5 billion ($2.5 billion per year) investment over 5 years [8]. To make this transformation a reality, national governments need to allocate the necessary funding and ensure that digital investments are part of their national plans and priorities. To justify and sustain such investments, there is an urgent need to increase the amount of available evidence on the impact, cost and cost-effectiveness of DHIs. Building this evidence base will require action from all stakeholders.

Donors supporting DHIs can offer funding for—or even require—analyses on impact, cost and/or cost-effectiveness as a standard part of the DHI. To ensure that monitoring and evaluation of DHIs is accessible to implementers, we need dedicated funding. In circumstances where there is a limited budget, modeling analyses based on high-quality data can be conducted to save precious funds.

Implementers need to prioritize and plan for impact and cost analyses as part of the DHI implementation to build community demand and acceptance, support country government ownership of the DHI and generate more funding for future DHIs. Implementers can do this even in the absence of a donor mandate by building internal capacity and using standardized tools, methodologies and resources.

Developers can also help generate impact evidence by building monitoring and evaluation tools into the intervention itself and ensuring that cost information is readily available. If developers want countries to implement their DHIs, they need to understand what is needed from country government decision-makers in order to do so.

Finally, country stakeholders can help by clearly communicating with implementing partners what specific information and evidence they need to see to make an informed decision on digital health investment or reinvestment. Additionally, they can include monitoring and evaluation assessments in their costed national digital health strategies. With all stakeholders working together—and with standardized, accessible methodologies—building the case for DHIs through impact and cost evidence is both feasible and necessary.

## Data Availability

The de-identified data underlying this article will be shared on reasonable request to the corresponding author.
